# Use of microbiology tests in the era of increasing AMR rates– a multicentre hospital cohort study

**DOI:** 10.1186/s13756-019-0480-z

**Published:** 2019-02-04

**Authors:** Brita Skodvin, Jannicke S. Wathne, P. Christoffer Lindemann, Stig Harthug, Roy M. Nilsen, Esmita Charani, Heidi Syre, Baard R. Kittang, Lars K. S. Kleppe, Ingrid Smith

**Affiliations:** 1 Norwegian Advisory Unit for Antibiotic Use in Hospitals, Department of Research and Development, Haukeland University Hospital, Haukelandsveien 22, 5021 Bergen, Norway; 2 Department of Clinical Science, University of Bergen, Jonas Lies vei 87, 5020 Bergen, Norway; 3 Department of Quality and Development, Hospital Pharmacies Enterprise in Western Norway, Møllendalsbakken 9, 5021 Bergen, Norway; 4 Department of Microbiology, Haukeland University Hospital, Haukelandsveien 22, 5021 Bergen, Norway; 5 Western Norway University of Applied Sciences, Inndalsveien 28, 5063 Bergen, Norway; 6 NIHR Health Protection Research Unit in Healthcare Associated Infections and Antimicrobial Resistance, 8th Floor Commonwealth Building, Imperial College London, Du Cane Road, London, W12 ONN UK; 7 Department of Medical Microbiology, Stavanger University Hospital, Gerd Ragna Bloch Thorsens gate 8, 4011 Stavanger, Norway; 8 Department of Medicine, Haraldsplass Deaconess Hospital, Ulriksdal 8, 5009 Bergen, Norway; 9 Department of Infectious Diseases and Unit for Infection Prevention and Control, Department of Research and Education, Stavanger University Hospital, Gerd Ragna Bloch Thorsens gate 8, 4011 Stavanger, Norway; 10 Department of Essential Medicines and Health Products, World Health Organization (WHO), Avenue Appia 20, 1211 Geneva 27, Switzerland

**Keywords:** Microbiology testing, Antibiotic prescribing, Antimicrobial resistance, Hospitals, Cohort study

## Abstract

**Background:**

Effective use of microbiology test results may positively influence patient outcomes and limit the use of broad-spectrum antibiotics. However, studies indicate that their potential is not fully utilized. We investigated microbiology test ordering practices and the use of test results for antibiotic decision-making in hospitals.

**Methods:**

A multicentre cohort study was conducted during five months in 2014 in Medical departments across three hospitals in Western Norway. Patients treated with antibiotics for sepsis, urinary tract infections, skin and soft tissue infections, lower respiratory tract infections or acute exacerbations of chronic obstructive pulmonary disease were included in the analysis. Primary outcome measures were degree of microbiology test ordering, compliance with microbiology testing recommendations in the national antibiotic guideline and proportion of microbiology test results used to inform antibiotic treatment. Data was obtained from electronic- and paper medical records and charts and laboratory information systems.

**Results:**

Of the 1731 patient admissions during the study period, mean compliance with microbiology testing recommendations in the antibiotic guideline was 89%, ranging from 81% in patients with acute exacerbations of chronic obstructive pulmonary disease to 95% in patients with sepsis. Substantial additional testing was performed beyond the recommendations with 298/606 (49%) of patients with lower respiratory tract infections having urine cultures and 42/194 (22%) of patients with urinary tract infections having respiratory tests. Microbiology test results from one of the hospitals showed that 18% (120/672) of patient admissions had applicable test results, but only half of them were used for therapy guidance, i.e. in total, 9% (63/672) of patient admissions had test results informing prescription of antibiotic therapy.

**Conclusions:**

This study showed that despite a large number of microbiology test orders, only a limited number of tests informed antibiotic treatment. To ensure that microbiology tests are used optimally, there is a need to review the utility of existing microbiology tests, test ordering practices and use of test results through a more targeted and overarching approach.

## Background

Effective use of microbiology test results has been shown to influence patient outcomes, health care costs and appropriateness of antibiotic prescribing and – use [1–3]. Microbiology tests have also for years provided antimicrobial resistance (AMR) surveillance data, informing empiric antibiotic therapy guidelines. With increasing AMR rates globally, sensitive, specific and affordable microbiology tests could be important tools in providing targeted antibiotic treatment to patients. The tests may facilitate de-escalation of antibiotic therapy from broad- to narrow spectrum treatment, thereby limiting the selection of drug resistant bacteria.

However, several studies indicate that the potential of microbiology tests is not fully utilized. Firstly, clinicians feel they cannot make full use of microbiology tests due to prolonged turnaround times (TATs) [4, 5]. Secondly, although many guidelines provide microbiology test ordering recommendations and information on how to interpret and use test results [6, 7], studies show that microbiology test ordering and use of test results are substandard [8–11]. As diagnostic microbiology methods evolve and become more sophisticated, these inadequacies may increase and ultimately result in incorrect antibiotic treatment for patients, as well as inefficient use of human and laboratory resources [12].

There are some studies on yield and utility of blood cultures, skin and soft tissue (SST) cultures, urinary pneumococcal antigen (UPAg) and polymerase chain reaction (PCR) tests detecting respiratory pathogens, and evidence exists for excessive ordering of urine cultures in asymptomatic patients [13–17]. However, there is little knowledge on existing microbiology test ordering practices and clinical use of microbiology test results, which is needed to optimize use of the tests. The aim of our study was therefore to investigate microbiology test ordering practices in hospitals and how microbiology test results were used to inform antibiotic decision-making. Our hypotheses were that a majority of current microbiology test ordering practices did not adhere to recommendations in the national antibiotic guideline and that a minority of microbiology test results were used to guide antibiotic treatment.

## Methods

### Design, setting and study population

This study was a multicentre cohort study conducted in infectious diseases-, gastroenterology- and pulmonary medicine wards across three emergency care and teaching hospitals in Western Norway. Patient data were originally collected for a multicentre cluster randomized controlled intervention study, evaluating antibiotic stewardship interventions in hospital settings [18].

Hospital A and B were tertiary care hospitals with 1100 and 600 beds, respectively, offering a full range of microbiology testing services. Hospital C was a secondary care hospital with 160 beds, referring the majority of microbiology specimens to hospital A. Infectious diseases- and pulmonary medicine wards were selected as these specialties have the highest consumption of antibiotics and thus order a large proportion of microbiology tests. Gastroenterology was included since hospital B had a joint medication storage area for the pulmonary medicine- and gastroenterology wards.

Microbiology test ordering practices were analysed using data from patients discharged from the study wards between February 10th and July 11th 2014. Only data from patients receiving antibiotic treatment for sepsis, urinary tract infections (UTIs), skin and soft tissue infections (SSTIs), lower respiratory tract infections (LRTIs) or acute exacerbations of chronic obstructive pulmonary disease (AECOPD) was included in the analyses. Patients admitted for < 24 h, > 21 days and/or readmitted within 30 days were excluded. Clinical use of test results was analysed for patients at hospital A, as complete microbiology test results were available at this hospital.

### Outcome measures

The primary outcome measures were microbiology test ordering practices and clinical use of microbiology test results. The secondary outcome measures were yield and TAT for the microbiology tests (Table 1).

**Table 1:** Outcome measures

Outcome	Description
Primary outcomes	
1: Microbiology test ordering practices	Measured by a. Degree of compliance with test ordering recommendations in the Norwegian national antibiotic guideline, by diagnoses [6]. b. Degree of microbiology test ordering, i.e. the proportion of patients who had different specimens obtained within the first three days after initiation of antibiotic treatment, by diagnoses and hospital sites
2: Clinical use of microbiology test results	The proportion of microbiology tests ordered on the day of admission used to guide antibiotic treatment. Use was assessed within the first two days after tests results were available to clinicians. For an antibiotic regime to be defined as adjusted in accordance with microbiology test result, it had to be susceptible to the identified pathogen and the regime least prone to drive antibiotic resistance. The evaluation took into account glomerular filtration rate and allergic reactions to antibiotics as recorded on admittance.
Secondary outcomes	
1: Yield of microbiology tests	The proportion of patients for which a specific test was positive and identified the potential causative pathogen. Reported by test and diagnoses.
2: Turnaround time for microbiology tests	Time in hours from the specimen was registered as received at the laboratories to final test results were available to clinicians in the electronic medical record. For blood cultures; time when gram stain results were made available to clinicians.

### Data collection

Patient data, including indication for antibiotic treatment, antibiotic treatment throughout the hospital stay, allergic reactions to antibiotics, glomerular filtration rate and number of days admitted were obtained from medical records and drug charts. Indications for antibiotic treatment were based on the treating physicians’ working diagnoses as recorded in patients’ medical records or drug charts on the day of initiation of antibiotic treatment. Laboratory data were collected from medical records to evaluate microbiology test ordering practices and yield, and from the laboratory information system to study clinical use of microbiology test results and TAT (Table 1). An overview of microbiology tests and test results are presented in Table 2. Bacterial cultures were identified by matrix assisted laser desorption ionization-time of light mass spectrometry (Maldi-Tof MS) and susceptibility testing was performed by disk diffusion tests or by minimum inhibitory concentration gradient tests. The PCR tests were developed in-house and the UPAg test was a lateral flow immunoassay.

**Table 2:** Overview of microbiology tests and test results

Microbiology tests	
Respiratory tests	Respiratory cultures Polymerase chain reaction (PCR) tests for viral and bacterial respiratory pathogens Urinary pneumococcal antigen tests
Skin and soft tissue cultures	Wound-, pus-, breastmilk- and/or tissue cultures
Blood cultures	
Urine cultures	
Test results	
Positive findings	Potential pathogen identified
a) Causative findings	Positive test results identifying causative pathogen
b) Non-causative findings	Positive test results reported as “contaminants”, “normal flora” or “mixed flora”
Negative findings	No pathogen identified

### Data analysis

Descriptive statistical analyses were performed on all outcome measures (Table 1). Chi-square test was applied to evaluate differences in microbiology testing practices between the hospitals, where the testing frequencies of each hospital were compared to the total test frequency of the two others. Fisher’s exact test was applied when numbers in one or more categories were < 5. Tests were two-sided and because of multiple testing, *p*-values < 0.01 were considered statistically significant. Statistical analyses were performed using the SPSS (Statistical Package for the Social Science) version 24.

## Results

In total, 1731 patient admissions were included in the analyses of microbiology test ordering practices. The mean age was 68 years old (range 15–103 years), the female/male ratio was 0.48/0.52, mean length of stay was 6.8 days and the 30 day-mortality rate was 8% (142/1731). The distribution of diagnoses was as follows: LRTI 35%, AECOPD 24%, sepsis 18%, SSTI 12% and UTI 11% (Table 3). Of the total patient cohort, 48% were recruited from Hospital A, 27% from Hospital B and 25% from Hospital C.

**Table 3:** Microbiology test ordering practices

			Microbiology test							
			Blood culture		Urine culture		SST culture^1^		Resp. test^2^	
Diagnosis^*^	Hospital		%	p^3^	%	p^3^	%	p^3^	%	p^3^
Sepsis	Hospital A	*n* = 205	95.1	0.64	70.2	0.02	10.7	< 0.01	60.0	< 0.01
	Hospital B	*n* = 42	88.1	0.06	76.2	0.81	2.4	0.34	78.6	< 0.01
	Hospital C	*n* = 73	97.3	0.38	86.3	0.01	1.4	0.02	17.8	< 0.01
	Total	*n* = 320	94.7		74.7		52.8		7.5	
UTI	Hospital A	*n* = 67	59.7	0.77	91.0	0.64	3.0	0.27	31.3	0.02
	Hospital B	*n* = 50	70.0	0.05	90.0	0.54	2.0	1.00	36.0	< 0.01
	Hospital C	*n* = 77	49.4	0.04	94.8	0.28	0.0	0.28	3.9	< 0.01
	Total	*n* = 194	58.2		92.3		1.5		21.6	
SSTI	Hospital A	*n* = 97	80.4	0.97	14.4	< 0,01	68.0	< 0.01	8.2	0.66
	Hospital B	*n* = 54	79.6	0.89	13.0	0.04	44.4	0.16	11.1	0.22
	Hospital C	*n* = 52	80.8	0.92	50.0	< 0,01	32.7	< 0.01	1.9	0.12
	Total	*n* = 203	80.3		23.2		52.7		7.4	
LRTI	Hospital A	*n* = 287	80.8	0.07	48.4	0.73	1.0	0.51	68.3	< 0.01
	Hospital B	*n* = 164	75.0	0.36	40.2	0.01	1.8	0.71	89.0	< 0.01
	Hospital C	*n* = 155	74.2	0.25	60.0	< 0,01	1.9	0.70	20.0	< 0.01
	Total	*n* = 606	77.6		49.2		1.5		61.6	
AECOPD	Hospital A	*n* = 172	74.4	< 0.01	38.4	0.42	2.3	0.46	59.3	0.29
	Hospital B	*n* = 152	57.9	0.01	33.6	0.02	1.3	1.00	92.1	< 0.01
	Hospital C	*n* = 84	63.1	0.54	58.3	< 0.01	1.2	1.00	14.3	< 0.01
	Total	*n* = 408	65.9		40.7		1.7		62.3	
All	Total	*n* = 1731	76.1		53.7		8.7		49.3	

*AECOPD: acute exacerbation of chronic obstructive pulmonary disease; LRTI: lower respiratory infection; SSTI: skin and soft tissue infection; UTI: urinary tract infection

^1^SST culture: wound, pus, breastmilk or tissue culture

^2^Resp. test: respiratory culture, polymerase chain reaction (PCR) test for viral and bacterial respiratory pathogens and/or urinary pneumococcal antigen test

^3^p value for testing whether there is a significant difference between one hospital compared to the total frequencies of the two others by Chi-square test or by Fisher’s exact test when numbers in one or more categories were < 5

### Guideline adherence

The degree of compliance with microbiology test ordering recommendations in the national antibiotic guideline was 89% across all diagnoses. Compliance was 95% in sepsis (blood culture), 92% in UTI (urine culture), 88% in LRTI (PCR test detecting respiratory pathogens, UPAg test, respiratory- or blood culture) and 81% in AECOPD (respiratory- and/or blood culture). There were no specific test ordering recommendations for SSTIs, however culture specimens were often obtained from the site of infection.

### Testing practices by tests, diagnoses and hospital sites

Many patients had more than one microbiology sample collected regardless of diagnosis (Table 3). In the total cohort of patient admissions, the following microbiology tests were ordered: 76% blood cultures, 54% urine cultures, 49% respiratory tests and 9% skin or soft tissue cultures. Among the patients with LRTI and AECOPD, 49 and 41% had urine cultures taken, respectively. Concomitantly, 22% of the patients with UTI had respiratory tests performed. Test ordering practices varied between the three hospitals. Patients diagnosed with sepsis, LRTI and AECOPD had significantly more respiratory tests taken at hospital B than at the two other hospitals (*p* < 0.01), and the same groups of patients had significantly less respiratory tests taken at hospital C compared to the two other hospitals (p < 0.01).

### Yield

The total yield for blood-, urine-, respiratory- and SSTI cultures was 8, 29, 34 and 67%, respectively (Table 4). For blood cultures, the yield was 20% in sepsis- and 4% in LRTI patients. For LRTI patients, the yield of the PCR test detecting respiratory pathogens, UPAg test and respiratory cultures was 18, 9 and 33%, respectively. However, 52% of the respiratory cultures had non-causative findings.

**Table 4:** Yield of microbiological specimen

Diagnosis*	Test findings	Blood culture %	Urine culture %	SST culture^1^%	Respiratory culture %	RP-PCR^2^ %	UPAg^3^ %
Sepsis	Causative	20	36	63	29	18	8
	Non-causative	5	16	25	58		
	Negative	75	48	13	13	82	92
	Total	100 (*n* = 303)	100 (*n* = 239)	100 (*n* = 24)	100 (n = 52)	100 (*n* = 106)	100 (*n* = 111)
UTI	Causative	17	54	33	0	0	0
	Non-causative	4	17	67	80		
	Negative	79	28	0	20	100	100
	Total	100 (*n* = 113)	100 (*n* = 179)	100 (n = 3)	100 (n = 15)	100 (*n* = 27)	100 (*n* = 21)
SSTI	Causative	4	30	70	40	0	0
	Non-causative	4	15	23	60		
	Negative	92	55	7	0	100	100
	Total	100 (*n* = 163)	100 (*n* = 47)	100 (*n* = 107)	100 (n = 5)	100 (n = 11)	100 (n = 4)
LRTI	Causative	4	16	67	33	17	9
	Non-causative	2	20	22	52		
	Negative	94	63	11	15	83	91
	Total	100 (*n* = 470)	100 (*n* = 298)	100 (n = 9)	100 (*n* = 185)	100 (*n* = 240)	100 (*n* = 196)
AECOPD	Causative	1	17	43	41	8	10
	Non-causative	4	16	43	45		
	Negative	94	67	14	14	92	90
	Total	100 (*n* = 269)	100 (*n* = 166)	100 (n = 7)	100 (*n* = 161)	100 (*n* = 167)	100 (*n* = 123)
Total	Causative	8	29	67	34	13	8
	Non-causative	4	18	25	51		
	Negative	88	53	8	14	87	92
	Total	100 (*n* = 1318)	100 (*n* = 929)	100 (*n* = 150)	100 n = (418)	100 (*n* = 551)	100 (*n* = 455)

*AECOPD: acute exacerbation of chronic obstructive pulmonary disease; LRTI: lower respiratory infection; SSTI: skin and soft tissue infection; UTI: urinary tract infection

^1^SST culture: wound, pus, breastmilk or tissue culture; ^2^RP-PCR: Respiratory panel polymerase chain reaction test for viral and bacterial respiratory pathogens; ^3^UPAg: Urinary pneumococcal antigen tests

### Turnaround time

Mean TAT was 25 h (95% CI, 22.4–27.7) for blood-, 37 h (95% CI, 31.2–42.6) for urine-, 56 h (95% CI, 49.5–63.0) for SST- and 80 h (95% CI, 60.5–99.6) for respiratory cultures.

### Clinical use of test results

**Fig. 1:**
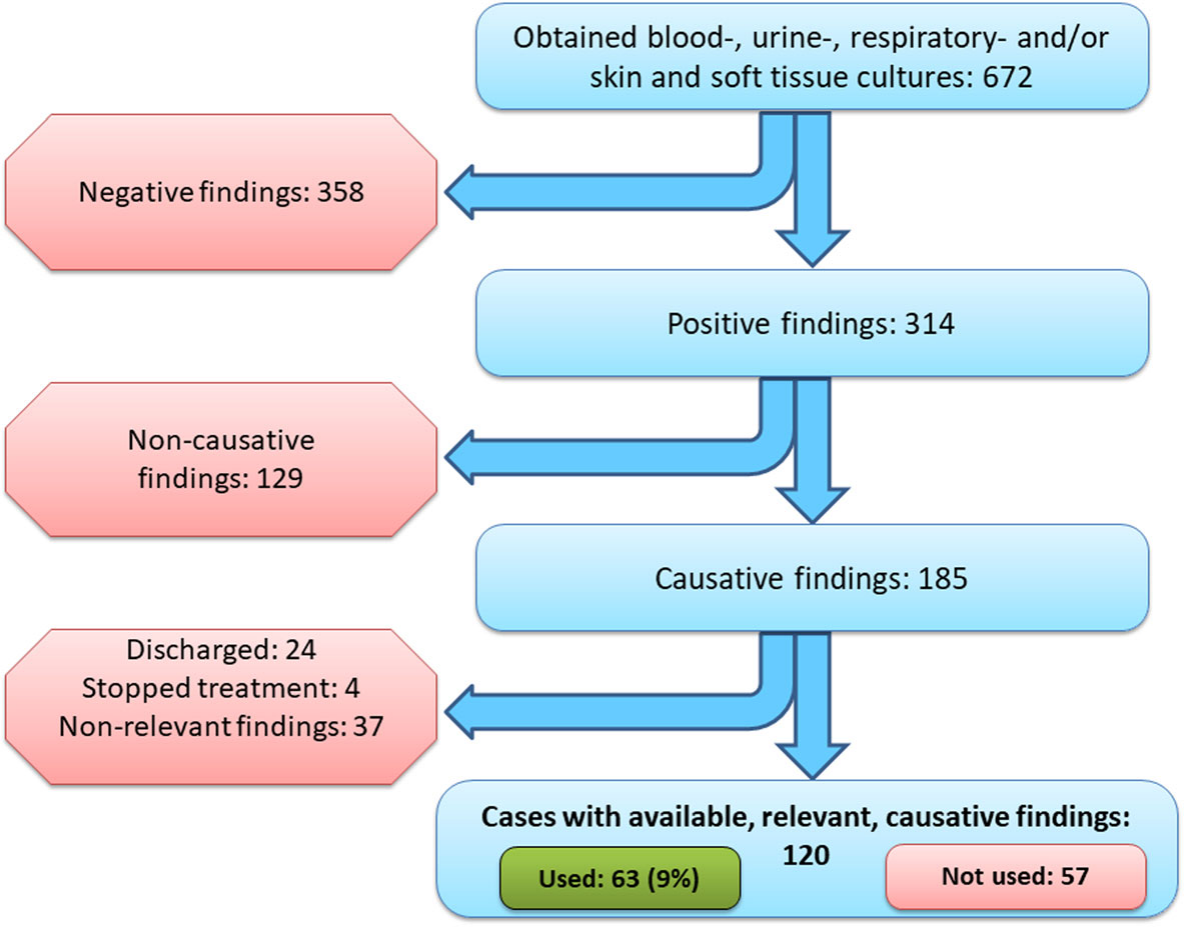
Patient admissions and use of microbiology test results

In hospital A, there were 828 patient admissions, of which 81 collected microbiology specimens at day > 1 after admission, leaving 747 cases eligible for inclusion in the analyses of clinical use of microbiology test results obtained on the day of admission. Of these, 672 (81%) had blood-, urine-, respiratory- and/or SST cultures taken and were included in the analyses of clinical use of test results (Fig. 1).

Of the 672 patient admissions, 358 (53%) had negative microbiology test results and 129 (19%) had non-causative findings. Among the remaining 185 cases, 37 had findings not relevant to their diagnoses, four had stopped antibiotic treatment and 24 were discharged when microbiology test results became available. Of the 120/672 (18%) inpatients with applicable findings, antibiotic treatment was adjusted according to test results only in 63 patients, i.e. 9% of the total number of patient admissions. Among the patients with the diagnoses SSTI and UTI, a majority had their antibiotic treatment adjusted in accordance with the test results, whereas treatment was adjusted only in a minority of the patients with AECOPD. As only 120 patient admissions had applicable test results, the number in each diagnostic group was low.

## Discussion

The main finding of this study was that despite a large number of microbiology test orders, only a small fraction of test results informed antibiotic decision-making. We observed high compliance with test ordering recommendations in the national guideline, but excessive testing across diagnoses, contributing to a low yield. TATs were long and microbiology test results with causative pathogens were underused, both contributing to the low utilization of the tests.

To our knowledge, this is the first study reporting on microbiology test ordering practices and use of test results in clinical practice. A previous study on clinical laboratory- and imaging tests, reported that one third of tests were unnecessary and only half of relevant test results were used in patient follow up [11]. Studies investigating the yield of blood culture and UPAg tests, showed similar results to ours [13, 14, 19, 20]. The high rates of respiratory cultures with non-causative findings identified in our material are also in accordance with the literature, reporting that respiratory sampling procedures are challenging [21].

Our study shows that the existing microbiology tests, testing practices and use of test results are not in accordance with the objective of microbiology testing; only a minor fraction of patients benefitted from a test result and only a small proportion could be used to target therapy and minimize the use of broad spectrum antibiotics. This suggests that microbiology laboratory resources could be spent more efficiently than producing insignificant or negative results, although negative microbiology test results may be important for treatment in some infectious disease patients. Only half of the patients with test results identifying the causative pathogen had their antibiotic treatment tailored accordingly, meaning that antibiotic treatment was not optimized for the other half of the patients.

There are several explanations for these findings. Excessive testing and inadequate follow up of test results may be caused by clinician’s insufficient knowledge of microbiology [22, 23]. Diagnostic uncertainty and inadequate routines for microbiology testing in the emergency departments may also contribute to the large number of unnecessary test orders [24]. However, the inherent characteristics of the microbiology tests play a major role for the yield; although adhering to the guidelines when sampling specimens, the yield for several tests was low. However, in this study, we did not have information on the quality of sampling and transportation of specimens, which may also impact the yield [8, 25]. Long TATs associated with certain tests may reduce their utility. We observed particularly long TAT for respiratory cultures. This may partly be explained by the time-consuming challenge of identifying and separating respiratory pathogens from normal bacterial flora. One reason for the continuation of these practices, both in the microbiology laboratories and in the clinical units, may be a lack of communication between the two parties to improve microbiology testing practices [23].

Our findings show the need for a systematic review of the use of microbiology tests in clinical practice. Firstly, tests with low yield should be evaluated, particularly tests for respiratory infections as our and other studies show that microbiology test results are of little help to identify causative pathogens [14, 20, 21]. Thus, more specific and sensitive tests in the diagnostic work up of respiratory infections are needed. Secondly, there is a need to review the indications for microbiology tests. Obviously, restricting urine cultures to patients with possible UTIs may reduce unnecessary antibiotic treatment of asymptomatic bacteriuria [26]. Additionally, although the overall yield for blood cultures was low in our study, it varied significantly between patients suffering from respiratory infections in the lower end and sepsis patients in the higher end. This indicates that a stratification, prioritizing blood cultures for the more severely ill patients, may be appropriate and increase overall yield.

Thirdly, there is a need to reduce TATs and increase the proportion of microbiology test results available at an early stage of patient treatment. Potential measures are expansion of molecular diagnostics, rapid and point-of-care test services, as well as revision of testing processes within the microbiology laboratories, shown to reduce TAT significantly [27, 28]. These measures, promoting rapid delivery of microbiology test results with better performance characteristics, are even more important in settings with higher rates of AMR than Norway [29]; In such settings, the identification of causative pathogens and their susceptibility to antibiotic agents, is crucial for appropriate and targeted antibiotic treatment. Furthermore, clinicians need to increase their knowledge of different microbiology tests; when to order them and how to apply the test results. Systematic measures such as providing education, audit with feedback on microbiology test ordering and use of test results, as well as establishing decision support for microbiology testing in computerized provider order entry systems, may be useful [30]. In order to accommodate all these challenges adequately, there is a need for clinical- and microbiology laboratory staff to work in partnership. Moreover, to develop sustainable and efficient solutions, there is a need for a targeted and overarching approach.

An improved utilisation of microbiology services is vital both for the individual infectious disease patients in need of optimised antibiotic therapy and for the containment of AMR. Microbiology tests can contribute to reduced use of broad spectrum antibiotics and antibiotics in general, thereby limiting the impetus for development and selection of drug resistant bacteria. With improved availability of microbiology test results and increased test accuracy, treatment can be more targeted and broad spectrum antibiotics saved [31]. Additionally, rapid access to microbiology test results differentiating viral and bacterial infections, may reduce unnecessary use of antibiotics [32].

In summary, this study raises several questions regarding the future of microbiology testing. How can we utilize microbiology testing and the laboratory resources more efficiently? Which diagnostic tests do we need to develop? And how can we improve interdisciplinary collaboration around the infectious disease patient? Thus, more research is needed on how to optimize the collection of microbiology samples, how to develop and implement new diagnostic methods and how to reduce TAT for microbiology tests, taking into account the potential impact on patient outcomes, antibiotic prescribing and development of AMR, as well as on use of human and laboratory resources.

The study has some limitations. Microbiology test results were mainly based on traditional culturing and Maldi-Tof MS. Use of novel technology such as molecular diagnostics could have decreased TAT and increased the proportion of test results used to inform antibiotic treatment [27]. Patient data used for analysis in this study were originally collected for an interventional study on antibiotic prescribing in hospitals [18]. However, we supplemented with microbiology data to accommodate the needs of this study. Data collection was limited to departments of internal medicine in Western Norway, potentially reducing the external validity. This is however a relatively large, multicentre study, applying an extensive amount of different data and covering a wide range of clinical scenarios.

## Conclusion

This study identified high compliance with microbiology testing recommendations in the national guideline. There was however extensive ordering of additional tests, many tests had low yield and only a small proportion of test results informed antibiotic decision-making. This highlights that the current use of microbiology laboratory services is suboptimal. There is a need both for tests with better performance characteristics and improved test ordering practices. Furthermore, use of microbiology test results to inform antibiotic decision-making needs to be optimized in order to ensure adequate patient treatment and more targeted therapy. To fill these gaps there is a need for an overarching approach with a clear call to fulfil the objective of microbiology testing; to provide rapid, sensitive test results to individual patients, but also to facilitate prudent use of antibiotics.

## Abbreviations

AECOPD: Acute exacerbation of chronic obstructive pulmonary disease
AMR: Antimicrobial resistance
LRTI: Lower respiratory tract infection
Maldi-Tof MS: Matrix assisted laser desorption ionization-time of light mass spectrometry
PCR: Polymerase chain reaction
SST(I): Skin and soft tissue (infection)
TAT: Turnaround time
UPAg: Urinary pneumococcal antigen tests
UTI: Urinary tract infection
